# Motor restrictions impair divergent thinking during walking and during sitting

**DOI:** 10.1007/s00426-021-01636-w

**Published:** 2022-01-08

**Authors:** Supriya Murali, Barbara Händel

**Affiliations:** Department of Psychology III, University of Würzburg, Würzburg, Germany

## Abstract

Creativity, specifically divergent thinking, has been shown to benefit from unrestrained walking. Despite these findings, it is not clear if it is the lack of restriction that leads to the improvement. Our goal was to explore the effects of motor restrictions on divergent thinking for different movement states. In addition, we assessed whether spontaneous eye blinks, which are linked to motor execution, also predict performance. In experiment 1, we compared the performance in Guilford’s alternate uses task (AUT) during walking vs. sitting, and analysed eye blink rates during both conditions. We found that AUT scores were higher during walking than sitting. Albeit eye blinks differed significantly between movement conditions (walking vs. sitting) and task phase (baseline vs. thinking vs. responding), they did not correlate with task performance. In experiment 2 and 3, participants either walked freely or in a restricted path, or sat freely or fixated on a screen. When the factor restriction was explicitly modulated, the effect of walking was reduced, while restriction showed a significant influence on the fluency scores. Importantly, we found a significant correlation between the rate of eye blinks and creativity scores between subjects, depending on the restriction condition. Our study shows a movement state-independent effect of restriction on divergent thinking. In other words, similar to unrestrained walking, unrestrained sitting also improves divergent thinking. Importantly, we discuss a mechanistic explanation of the effect of restriction on divergent thinking based on the increased size of the focus of attention and the consequent bias towards flexibility.

**Supplementary Information:**

The online version contains supplementary material available at 10.1007/s00426-021-01636-w.

## Introduction

The notion that walking and thinking are linked goes back to the Peripatetic school of philosophy in ancient Greece and throughout history, there have been anecdotal claims that walking helps people think, solve problems and come up with creative ideas. Over the past couple of decades, some empirical evidence for these claims has been obtained.

Creativity has been proposed to involve two main processes: divergent and convergent thinking (Guilford, 1967). Divergent thinking, which is the focus of this study, is the ability to come up with new ideas or solutions to a single problem. Convergent thinking, on the other hand, involves the generation of a single novel solution by bringing together different concepts or problems. Two of the most commonly used tests are Guilford’s alternate uses test or AUT (Guilford, 1967) for divergent thinking and Mednick’s remote association test or RAT (Mednick, 1962) for convergent thinking. In the AUT, participants are asked to come up with alternate uses for everyday objects and in the RAT, they are required to find a commonality between three given words. Although these tests assess specific processes, they have been said to be good indicators for creative potential (Runco & Acar, 2012). With regard to the AUT, there are several sub-scores, such as fluency, flexibility and originality. In this study we use fluency and flexibility sub-scores of the AUT, which is defined by the number of valid responses generated and the number of categories the responses can be assigned to, respectively, to characterize divergent thinking.

Concerning large body movements, walking has been experimentally associated with better performance in divergent thinking (Kuo & Yeh, 2016; Leung et al., 2012; Oppezzo & Schwartz, 2014; Zhou et al., 2017). For instance, Oppezzo and Schwartz (2014) found that people performed better in a divergent thinking task while walking as opposed to sitting. The same authors also found that there was a residual effect of walking by showing that performance continued to be enhanced when people performed the sitting condition after the walking condition.

Specifically, not just walking, but free walking has been shown to have the most benefit for divergent thinking. Kuo and Yeh (2016) showed that performance in the AUT improved during unrestrained or free walking as opposed to walking in a prescribed rectangular path. In the same study, the authors tested whether the effect was due to the path itself by including a condition, where participants had to walk in a path which was generated during another participant’s free walking condition. They found that performance improved only during the actual free walking and thus concluded that it is the freedom to move and not the characteristics of the path that has an influence.

Although unrestricted walking in particular seems to have an effect, to our knowledge, no study has tested if this effect pertains to motor restriction in general. However, studies have suggested that free walking as well as other fluid movements can form an association of bodily states and abstract concepts and thereby improve divergent thinking (Kuo & Yeh, 2016; Leung et al., 2012; Slepian & Ambady, 2012, 2014). Slepian and Ambady (2012), for instance, found that AUT fluency scores were higher when participants made fluid movement with the hand while drawing as opposed to when they made non-fluid movements (by drawing straight lines). The authors argue that fluid movements could be linked to fluid thought processes. A similar argument was made by Kuo and Yeh (2016), namely, that walking/moving freely could activate the idea of mind wandering.

Albeit less, there also is evidence for a link between creativity and eye-related movements, namely, blinks. Eye blinks are of three types: reflexive, spontaneous or voluntary. Reflexive blinks occur as automatic responses to startling external stimuli, such as an air puff. Voluntary blinks occur when someone is explicitly asked to make a blink. Spontaneous blinking, which is the focus of our study and has been found to be linked with divergent thinking (Akbari Chermahini & Hommel, 2010; Ueda et al., 2016), is blinking that occurs unconsciously and not as a reflexive response. These blinks occur about 10–15 times a minute (Burr, 2005; Doughty, 2001; Kaminer et al., 2011). Although they serve to moisten the eyes, their frequency is much higher than what is required just for that purpose (Kaminer et al., 2011). The purpose of these surplus blinks is not completely decoded, but they have been shown to reflect cognitive processes (Brych & Händel, 2020; Brych et al., 2020, 2021; Fogarty & Stern, 1989; Goldstein et al., 1992). One study by Akbari Chermahini and Hommel (2010) found that participants who had a comparably moderate baseline eye blink rate, as identified as the centre of the distribution of blink rates for this specific study, had higher scores on the AUT compared to subjects showing blink rates at the left and right side of the distribution. Another study, specifically focussing on blinking during the task as opposed to during a baseline period, found a positive correlation between blink rate and AUT scores (Ueda et al., 2016).

Despite this sparse evidence from directly testing for creativity, there is rather strong experimental indication that cognitive aspects that might be applied during creative thinking (Madore et al. 2015) are linked to eye movements. When people engage in imagination and memory retrieval, they tend to make more saccades and blinks (Salvi & Bowden, 2016). Although eye blinks are not specifically investigated, a number of studies have found a link between eye movements in general and memory retrieval (Damiano & Walther, 2019; Johansson & Johansson, 2014; Johansson et al., 2012; Lenoble et al., 2019). In fact, not allowing any eye movements worsens performance in these tasks (Damiano & Walther, 2019; Johansson & Johansson, 2014). Moreover, Johansson and Johanssonn (2014) also showed that asking participants to make eye movements that were incongruent with the position of the objects during a visuospatial memory task, worsened performance as opposed to congruent movements. Apart from visual memory, a study by Lenoble et al. (2019) showed that eye movements could also aid in retrieving autobiographical memories. These authors found memories retrieved while making free eye movements were more detailed and faster than those retrieved during fixation. Moreover, Salvi and Bowden (2016) in their paper, mention how it has been anecdotally suggested that closing one’s eyes is thought to reflect the process of disengaging from the outside world and concentrating on inner thoughts. Indeed, we recently showed that blinks could directly reflect cognitive processes independent of external sensory input (Brych & Händel, 2020; Brych et al., 2021; Murali & Händel, 2021).

Therefore, while there is strong evidence that eye movements are related to cognitive processes and some evidence that they are linked to creativity, it is not clear if eye movements play a role in cognition or if they are linked via a common process. Akbari Chermahini and Hommel (2010) suggested that the link between eye blinks and divergent thinking performance could be mediated by dopamine levels. Dopamine has been shown to play a role during divergent thinking (Kulisevsky et al., 2009; Zabelina et al., 2016) and at the same time is correlated with eye blink rates (Bologna et al., 2012; Karson, 1983; Taylor et al., 1999). Blink rate has been shown to increase following the administration of dopamine agonists (Karson et al., 1984) and decrease following dopamine antagonists (Blin et al., 1990; Strakowski & Sax, 1998; Strakowski et al., 1996). However, a few recent studies have found a lack of evidence for the positive relationship between dopamine and eye blinks in healthy human adults (Dang et al., 2017; Sescousse et al., 2018). Overall, when it comes to eye blinks, dopamine and creativity, the relationship is hard to interpret, since there are several neuronal processes modulated by dopamine, which could influence blinking as well as creativity. For example, a link might be realized via the default mode network. Blinks activate the default mode network (Nakano et al., 2013), which has been shown to play a role in creativity (Beaty et al., 2014; Kühn et al., 2014) as well as to be linked to dopamine (Dang et al., 2012; Nagano-Saito et al., 2009).

Using three experiments, we tested the influence of body movement and motor restriction on the AUT and additionally tested the relationship between blinks and performance during the different conditions and the interaction between blinks and other body movements. In experiment 1, we focused on the interaction between walking and blinking. When investigating specific movements, it is important to consider that many body movements interact. As shown by us and by others, spontaneous blink rate increases can be linked to movements of the mouth during speech (Brych et al., 2020; von Cramon & Schuri, 1980). In addition, walking goes hand in hand with an increased blink rate (Cao et al., 2020). Importantly, this association is not dependent on the visual input, as it persists even during absolute darkness, suggesting a link deeply integrated into the system. We, therefore, set out to better understand the influence of different movement states on creativity, while additionally assessing the interactions between large movements and blinks. The purpose of experiment 1 was to (a) replicate previous findings on walking and blinking on divergent thinking, (b) to differentiate the effects for the different phases of the creativity task (baseline, thinking, responding), and (c) to understand if effects of blinking and walking are additive or independent from each other.

The aim of experiment 2 was to understand if the mere execution of motor output is the relevant factor or if it rather is the specific instruction/ body state, which introduces the effect. As detailed earlier, particularly walking without restriction to the path, has been shown to improve divergent thinking. Free eye movements, although not been studied in the context of creativity, have been shown to benefit memory. We wanted to understand if the positive effect on cognition extends to other movement states and hence, compared performance in the AUT not only during free and restricted walking, but additionally during free and restricted sitting. The aim of experiment 3 was to replicate the findings of experiment 2 and importantly test if eye blinks show a link to fluency depending on the level of restriction.

## Experiment 1

### Participants

Twenty fluent German speakers (7 males) between the ages of 18 and 35, took part in the first experiment. All participants were bachelor’s students from the Psychology department at the University of Wuerzburg and were recruited via the SONA-systems software. They gave their written informed consent and received study credit for their participation. The study complied with the European data protection law (DSGVO).

### Procedure

#### Stimulus and equipment

We used 14 words (translated to German) from the Guilford’s Alternate Uses Task: bandage, brick, chair, desk, frying pan, garbage bag, lipstick, pencil, newspaper, shoes, spoon, tile, toothbrush, and towel. These specific words were chosen based on two previous studies (Akbari Chermahini & Hommel, 2010; Ueda et al., 2016). In particular we used 13 words from Ueda et al. (2016) and additionally added the word “newspaper” from Akbari Chermahini and Hommel (2010) to get an even number of words to divide among the two conditions. A headset (Sennheiser PC3) was used to present the words and to record the verbally given responses. The words as well as the instructions (more details in the next section) were presented via a text-to-audio function in MATLAB using an automated voice for audio output. The verbally given responses were saved as a raw audio (wav) format. A response key connected via a response box (model: K-RB1-4; The Black Box ToolKit Ltd, UK) was provided and used by the participant to begin the trials or conditions whenever they were ready (see next section for more information). The experimental program was implemented in MATLAB 2015a, with the Psychophysics Toolbox extensions (Brainard, 1997; Kleiner et al., 2007; Pelli, 1997), using a Dell Precision (M6700) laptop running Windows 10. Binocular eye movements were recorded using the 120 Hz SMI video-based eye tracking glasses (SensoMotoric Instruments GmbH, Berlin, Germany). The experiment was conducted in a normally lit room of size 9 m*2 m and took approximately 90 min.

#### Experimental design

A within-subject design was applied to test the effects of the two conditions: walking vs. sitting. The conditions were presented in two blocks, each consisting of seven trials (words), with the order of the blocks randomized between subjects. Specifically, at the start of the experiment, a randomization was applied, such that a random order for the conditions and words was generated. Note that 7 subjects did the walking condition first and 13 subjects did the sitting condition first.

Before starting the experiment, subjects were prepared with the eye tracker, the headset and the laptop in a backpack. The experimenter then gave the initial instructions as to the task and the conditions. Participants were informed that they would have a minute to think after each word is presented. They were asked to give as many ideas as possible and told that they should not give non-uses, such as ‘throw away’. After this, subjects could press a key to receive further instructions and information regarding which condition they were going to start with, through an automated voice via the headphones.

Before starting each condition, the automated voice indicated that the baseline would begin and that an auditory beep would mark the start and end of the baseline period. Subjects were instructed to either walk or sit during this baseline, depending on the condition. No external stimulus or task was given during this period. In the walking condition, subjects were allowed to walk freely around the room. In the sitting condition, no specific instructions were given except that the subject had to comfortably sit in a chair. After the baseline, the audio instructions would again indicate that a trial was about to start and that a word would be presented followed by two auditory beeps to indicate the start and end of think time, respectively. After the think time, participants were asked to give their responses after another beep. Finally, participants were informed of the end of the (3 min) response time and that they could take a break and press a key for the next trial (or condition) to start. Note that the procedure for a trial with a separate think time and response time was similar to Ueda et al. (2016). The reason for this was because voluntary actions such as speaking is associated with an increase in blinking (Brych et al., 2020; von Cramon & Schuri, 1980). Participants were allowed breaks between trials and between conditions. However, there was no minimum or maximum time between conditions and all measurements were completed in a single session.

### Scoring of the AUT

We scored the responses using the criteria from a previous study (Ottemiller et al., 2014). We first took all responses given by the subject and then filtered out responses that were (1) repetitive (a direct repetition of a previous use given); (2) implausible (given the objects properties, e.g., pen used as a skirt) or (3) a non-use (e.g., throw in the garbage). For the repetition criteria, only direct repetitions were used. For example, for the object “towel”, responses such as “use as a skirt” and “use as a t-shirt” were considered separate ones. However, responses such as “used as shirt” and “used as a blouse”, were regarded as one idea, whether they were given consecutively or not. Responses that might belong to the same category (in the case of the first example: clothing) were not treated as repetitions. However, responses belonging to a specific subcategory (in the case of the second example: tops) were considered repetitions. This was done to avoid high fluency scores stemming from giving random repetitive ideas (Zhang et al., 2020).

Fluency was then defined as the total number of correct responses and flexibility was defined by the different categories in the responses.

### Blink detection

We used a blink detection algorithm based on the pupil radius data similar to our previous studies (Brych & Händel, 2020; Brych et al., 2021; Murali & Händel, 2021). Blinks were detected if the z-transformed radii data of either of the two eyes was missing or if the data from one of the eyes was missing and the other eye had a *z* value below a certain threshold (-1, -2 or -3 set for each individual subject). These blinks were then extended until the radii data of either eye was higher than the set threshold. Blinks that were less than 100 ms apart were combined and those longer than 0.5 s were discarded.

### Results

#### AUT fluency and flexibility score during walking vs. sitting

**Fig. 1:**
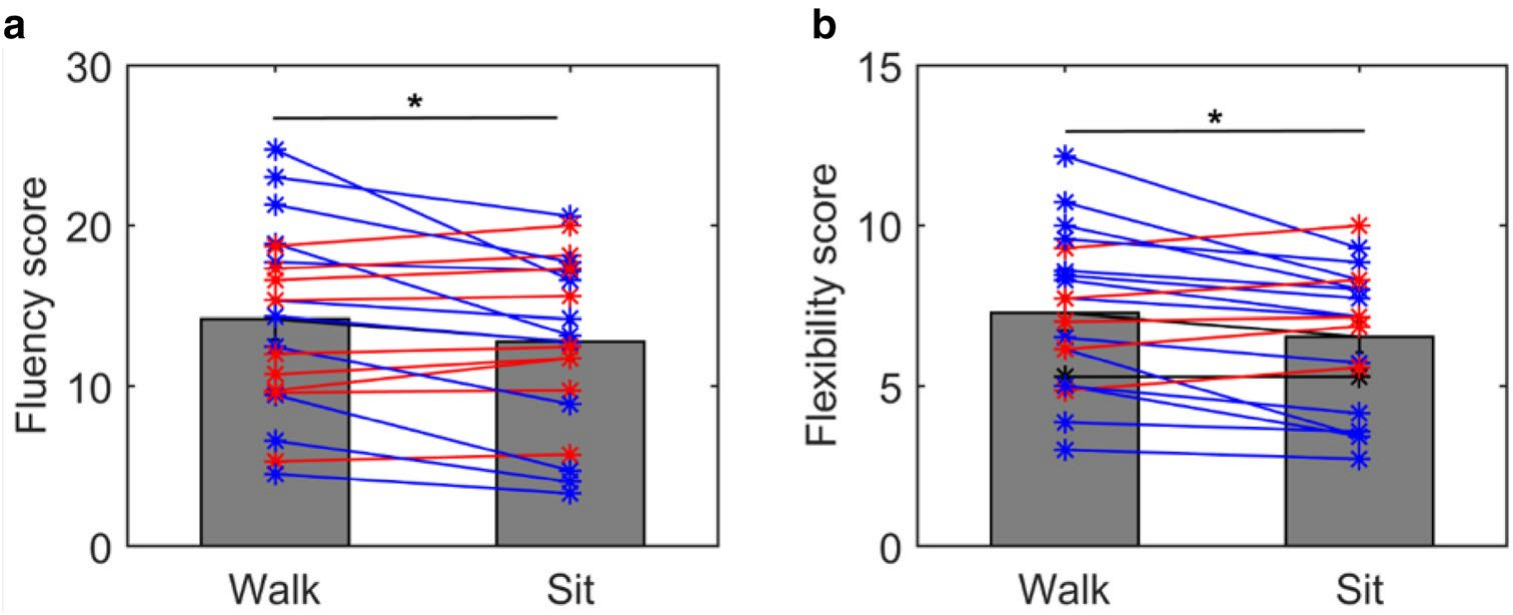
Mean fluency score (**a**) and the mean flexibility score (**b**) during walking and during sitting are shown. *T* tests revealed a significant difference between walking and sitting for fluency (*t*(19) = 2.4; *p* = 0.03) and flexibility (*t*(19) = 2.9, *p* = 0.008). The asterisk symbols represent the data from each subject. The blue lines represent cases, where the fluency score is higher for the walking condition and the red lines represent cases, wherein it is higher for the sitting condition

The mean fluency score during walking was 14.1 (SD = 5.7) correct responses per trial and during sitting was 12.7 (SD = 5.3) correct responses per trial (see Fig. 1). The mean flexibility score during walking was 7.3 (SD = 2.4) and during sitting was 6.5 (SD = 2.1). A *T* test revealed a significant difference between the two conditions for fluency (*t*(19) = 2.4; *p* = 0.03) and for flexibility (*t*(19) = 2.9, *p* = 0.008). In addition, Supplementary Fig. S2 shows the mean fluency score over all subjects for each word. A within subject ANOVA revealed no significant effect of the words (*F*(13,269) = 0.96, *p* = 0.5).

#### AUT fluency and flexibility score vs. eye blink rate

**Fig. 2:**
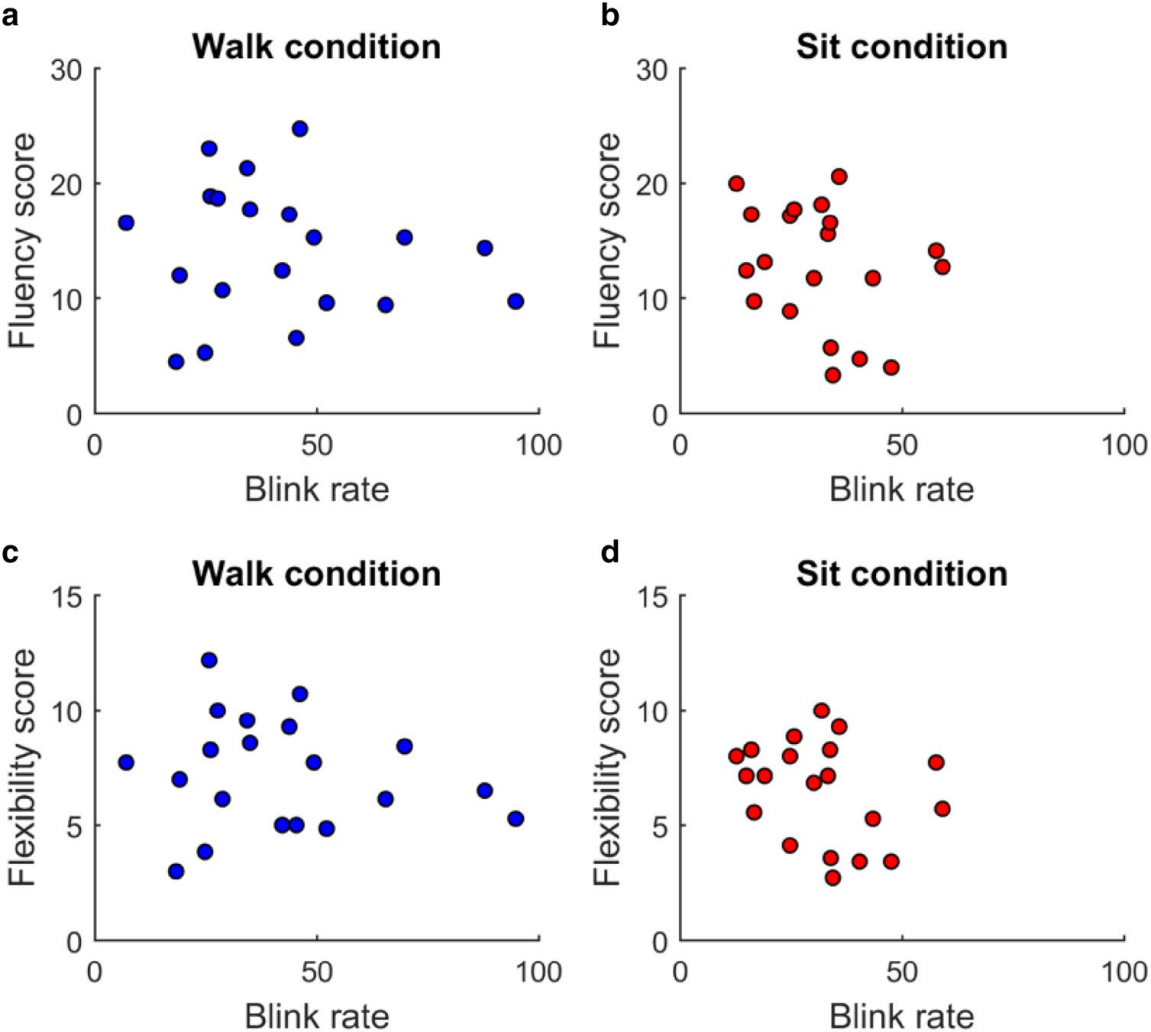
Mean fluency score (**a**, **b**) and flexibility score (**c**, **d**) is plotted against the blink rate for each subject during walking (**a**, **c**) and during sitting (**b**, **d**). Each coloured circle represents data from a single subject. Linear regression gave no indication that eye blinks predicted the fluency or flexibility scores during the think time

The mean think-time eye blink rate (blinks per minute) during walking was 42.1 (SD = 22.9) and during sitting was 31.7 (SD = 13.2). A linear regression model was applied to see if eye blink rates (during the think time) predicted the fluency and flexibility scores (see Fig. 2). For fluency, the analysis did not reveal any significance for either the walking (*F*(1,19) = 0.2, r2 = 0.01, *p* = 0.7) or the sitting (*F*(1,19) = 0.001, r2 = 4.49e−05, *p* = 0.7) condition. In addition, for flexibility, there was no significance either in the walking (*F*(1,19) = 0.2, r2 = 0.01, *p* = 0.6) or the sitting (*F*(1,19) = 0.07, r2 = 0.004, *p* = 0.7) condition.

#### Eye blink during walking vs. sitting and task vs. no task

To see if eye blink rates differed during the conditions and the different phases of the trial, we conducted a repeated measures two-factor ANOVA for the factors condition (Walk vs. Sit) and task phase (Baseline vs. Think time vs. Response Time).

**Fig. 3:**
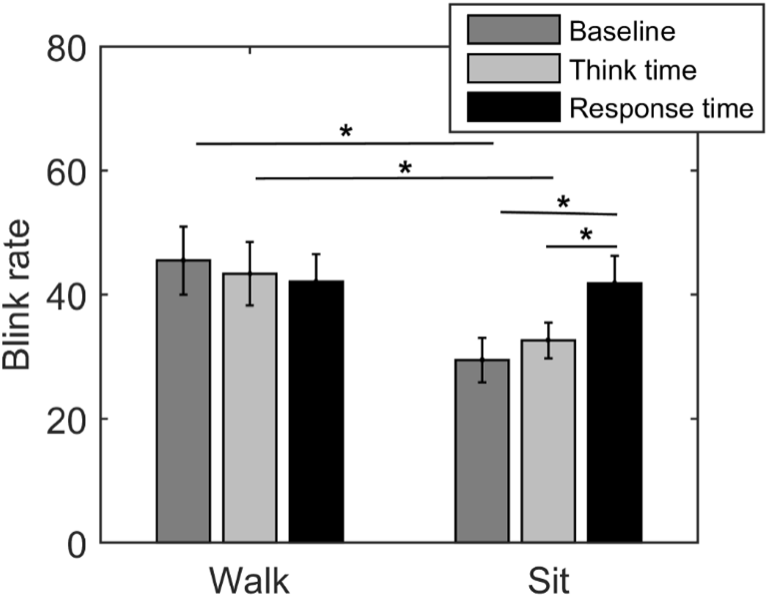
Mean blink rate during the different phases of the task, namely, baseline, think time and response time, for the walking and sitting condition is shown. A two-factor ANOVA revealed a significant effect of condition (*F*(1,125) = 9.95, *p* = 0.005) and a significant interaction (*F*(2,125) = 4.05, *p* = 0.025), but no significant effect of the different phases of the task (*F*(1,125) = 0.86, *p* = 0.43)

There was a significant effect of condition (*F*(1,125) = 9.95, *p* = 0.005). However, no significant effect of task phase (*F*(1,125) = 0.86, *p* = 0.43). From Fig. 3, it is clear, that eye blink rates during walking were higher than during sitting. In addition, it can be seen that task phase indeed has an effect on blink rate but only when sitting. This is confirmed by a significant interaction effect (*F*(2,125) = 4.05, *p* = 0.025).

Post-hoc *T* tests showed that eye blink rates were higher for baseline walking than baseline sitting (*t*(20) = 2.5, *p* = 0.02), as well as for think-time walking compared to the think-time sitting (*t*(20) = 2.7, *p* = 0.01), but not for response time walking compared to response time sitting (*t*(20) = 0.3, *p* = 0.8). In fact, eye blink rate during the sitting response time was higher than the sitting baseline (t (20) = 2.7, *p* = 0.01) and the sitting think time (*t*(20) = 2.8, *p* = 0.01).

#### Interim discussion

Experiment 1 showed that performance in the AUT is higher when walking than when sitting. However, as can be seen in Fig. 1a, b, nine participants in the fluency score and five in the flexibility score showed the opposite trend. Interestingly, it seems that participants showing an improvement when walking do so rather pronounced, whereas those participants who are better for sitting, are only slightly better. It is important to point out that this is not caused by the order of conditions, as walking always showed a groupwise averaged improvement in fluency compared to sitting, independent if people walked or sat first (Fig. S1). Nevertheless, in a supplementary analysis the improvement only reaches significance for those participants who sat first, which could be indicative of a residual effect of walking, as described in the literature (Oppezzo & Schwartz, 2014). However, as there is a power difference between walking first (*N* = 7) and sitting first (*N* = 13), a larger sample size would have been necessary to draw conclusions as to such a residual effect.

As to the reason why not all subjects showed an improvement in AUT performance for walking compared to sitting, we speculate that the difference in movement restriction caused this division. As restriction was not explicitly instructed for experiment one, it is possible that some subjects interpreted the sitting condition as an unrestricted condition in which they moved freely (while sitting), whereas the other group of subjects interpreted the requirement of the sitting condition as moving as little as possible. This might explain why one group was very comparable in its performance (walking and sitting was equally unrestricted), whereas the other group showed gross difference in performance in the unrestricted walking condition as opposed to a very restricted baseline. In experiment 2 we directly tested if the effect of improved AUT performance is mediated by restriction, independent of walking.

## Experiment 2

### Participants

Seventeen new participants who were fluent German speakers (4 males) between 18 and 35 years, took part in the second experiment. They received monetary compensation for their participation. The study complied with the European data protection law (DSGVO) and was additionally approved for Hygiene regulations regarding COVID-19. All participants were recruited via the SONA-systems software.

### Procedure

#### Stimulus and equipment

We used 12 words out of the 14 translated words that were used in experiment 1: bandage, brick, chair, desk, frying pan, garbage bag, lipstick, pencil, newspaper, shoes, spoon, tile. Similar to experiment 1, the experimental program was implemented in MATLAB 2015a, with the Psychophysics Toolbox extensions (Brainard, 1997; Kleiner et al., 2007; Pelli, 1997), using a Dell Precision (M6700) laptop running Windows 10. We also used the same response key connected to a response box (model: K-RB1-4; The Black Box ToolKit Ltd, UK). Restrictions due to the COVID-19 pandemic did not permit the use of an eye tracker or headset. All instructions and the words were instead presented directly using the in-built speakers of the laptop. This was again done similar to experiment 1, using a text-to-audio function in MATLAB. The responses were recorded via the in-built microphone and saved as a raw audio recording (.wav format). The experiment was conducted in a normally lit room of approximately 5*6 m^2^ and took approximately 60 min.

#### Experimental design

Similar to experiment 1, a within subject design was conducted with four conditions and three words (trials) per condition. At the start of the experiment, a randomization was applied, such that a random order for the conditions and words was generated. The order of the conditions for each subject is presented in the Supplementary Table S1. Note that all subjects completed all condition and the recording was done in a single day. Similar to experiment 1, participants were allowed breaks in between conditions and trials, with no minimum or maximum length.

Before starting each experiment, subjects were informed by the experimenter as to the task and conditions. They were instructed that they would have 1 min to think about the answers and 3 min to give their answers and that they could give as many responses as possible, but should not give non-use responses, such as “throw away”. During this initial instruction phase, participant were also shown the restricted path that they had to take (details below).

Once the experiment began, the automated voice indicated the condition and that a trial was about to start. Note that unlike experiment 1, no baseline measurement was conducted, since no eye tracker was used and the main purpose of the baseline in experiment 1 was to measure baseline eye blink rates. At the start of each trial, similar to experiment one, the audio instructions would indicate that a word would be presented followed by two beeps to indicate the start and end of the 1 min think time. Following the end of the think time, participants were asked to start giving their responses after another beep. At the end of the (3 min) response time, participants were allowed to take a break and could press a key whenever they were ready to go to the next trial or condition. The experimenter kept a close watch on the participant, to detect obvious violations of the task requirements with regard to movement or restriction.

The four conditions were as follows:

1. *Free walking*: subjects could walk around a large room without any restriction as to the path.
2. *Restricted walking*: subjects had to walk back and forth in a straight path from wall to wall in the centre of the room. A horizontal mark depicted the width of the path.
3. *Free sitting*: subjects sat comfortably on a stationary non-rotating armchair with a solid non-moving back and no wheels. For all subjects the position of the chair was against one of the walls of the room such that the person would face the room and not the blank wall.
4. *Restricted sitting*: subjects sat at a distance of 50 cm from a computer screen with a fixation cross at the centre. The position of the chair and laptop was always the same for all subjects. As we did not track motor output, we cannot assess to what extent subjects were moving in the sitting condition.

### Scoring of the AUT

We used the fluency and flexibility sub-scores as in experiment 1. The criteria were the same with the following responses considered as incorrect (1) repetitive (a direct repetition of a previous use given); (2) implausible (given the objects properties, e.g., pen used as a skirt) or (3) a non-use (e.g., throw in the garbage). For more details on the exclusion criteria, please see experiment 1.

### Results

#### Effect of movement and restriction on fluency and flexibility

**Fig. 4:**
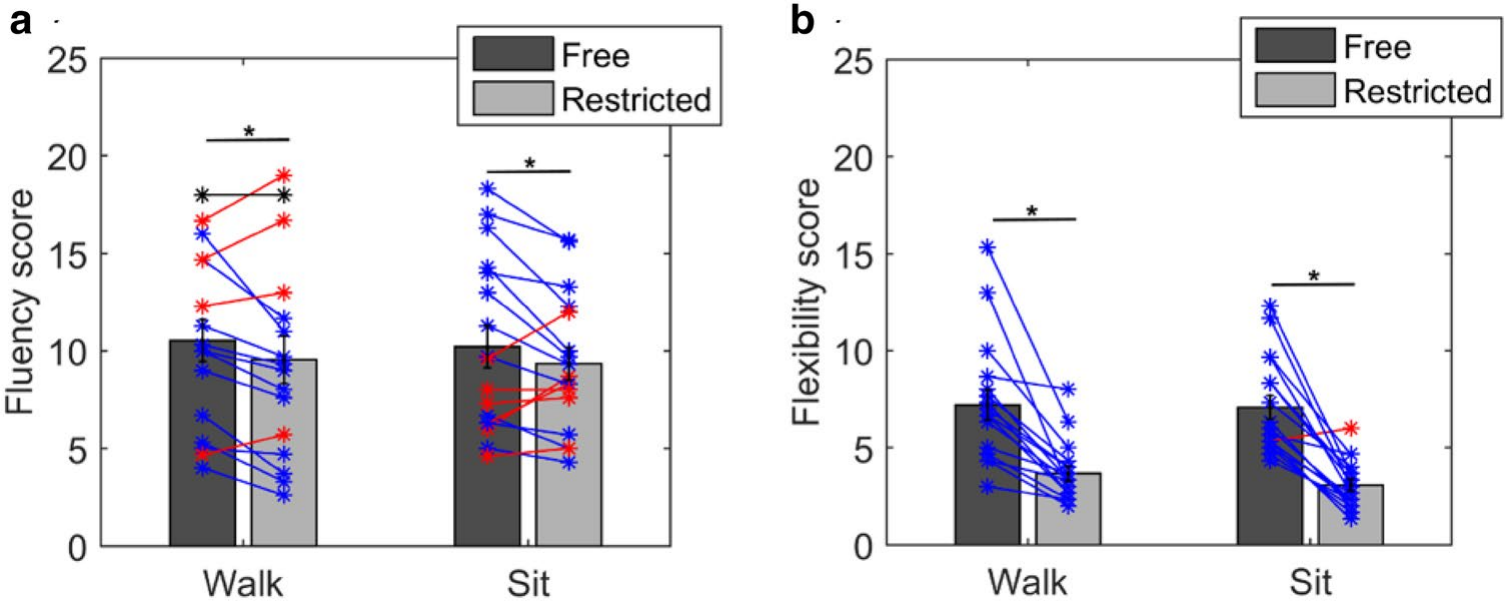
Mean fluency score (**a**) and the mean flexibility score (**b**) during free and restricted walking and sitting are shown. For the fluency, a two-factor ANOVA revealed a significant effect of restriction (*F*(1,67) = 8, *p* = 0.01), but not movement (*F*(1,67) = 0.35, *p* = 0.56), and no significant interaction (*F*(1,67) = 0.02, *p* = 0.8). Similarly, for flexibility, the two-factor ANOVA showed a significant effect of restriction (*F*(1,67) = 39.4, *p* < 0.001) but no significant effect of movement (*F*(1,67) = 1.07, *p* = 0.3) and also no significant interaction (*F*(1,67) = 1.6, *p* = 0.2). The asterisks represent data from individual subjects. The blue lines represent cases with higher scores during free movement, the red lines represent higher scores during restriction and black lines represent equal scores

The mean fluency score during free walking was 10.5 (SD = 4.4), during restricted walking was 9.5 (SD = 4.9), during free sitting was 10.2 (SD = 4.4) and during restricted sitting was 9.3 (SD = 3.5). The mean flexibility score during free walking was 7.2 (SD = 3.2), during restricted walking was 3.6 (SD = 1.6), during free sitting was 7.1 (SD = 2.5) and during restricted sitting was 3.1 (SD = 1.2). Figure 4 shows the mean fluency and flexibility scores during all four conditions.

For fluency, a repeated-measures two-factor ANOVA was conducted between the factors movement (Walk vs. Sit) and Restriction (free movement vs. restricted movement). The results showed a significant effect of restriction (*F*(1,67) = 8, *p* = 0.01), but not movement (*F*(1,67) = 0.35, *p* = 0.56), and also no significant interaction (*F*(1,67) = 0.02, *p* = 0.8). A post-hoc *T* test for the factor Restriction (free movement vs. restricted movement) revealed that fluency scores were significantly higher during free movement (*t*(33) = 2.8, *p* = 0.01). Similarly, for flexibility, the two-factor ANOVA showed a significant effect of restriction (*F*(1,67) = 39.4, *p* < 0.001) but no significant effect of movement (*F*(1,67) = 1.07, *p* = 0.3) and also no significant interaction (*F*(1,67) = 1.6, *p* = 0.2). A post-hoc *T* test for the factor restriction (free movement vs. restricted movement) revealed that fluency scores were significantly higher during free movement (*t*(33) = 8.7, *p* < 0.001).

Supplementary Fig. S2 additionally shows the mean fluency score over all subjects for each word. Note that, just as in experiment 1, a within-subject ANOVA revealed no significant effect of the words (*F*(11,203) = 0.3, *p* = 0.9).

#### Interim discussion

Experiment 2 showed that the absence of movement restriction rather than walking per se improves performance in the AUT. With this insight, we conducted a third study to (i) replicate the importance of restriction and (ii) to examine the relationship between blink rates and fluency or flexibility scores in dependence of restriction.

## Experiment 3

### Participants

Twenty-three new participants, who were fluent German speakers (4 males) between 18 and 35 years, took part in the third experiment. However, one subject had to be excluded for the eye blink analysis, because the eye tracker had a technical problem during the recording. All participants received monetary compensation for their participation. The study complied with the European data protection law (DSGVO) and was additionally approved for Hygiene regulations regarding COVID-19. All participants were recruited via the SONA-systems software.

### Procedure

#### Stimulus and equipment

We used 8 words in this experiment: bandage, brick, chair, desk, frying pan, garbage bag, lipstick, newspaper. For details regarding the experimental room and equipment, please refer to experiment 2. Binocular eye movements were recorded using Pupil Core mobile eye tracker developed by Pupil Labs (Kassner et al., 2014). The experiment took approximately 120 min.

#### Experimental design

Similar to experiment 2, a within subject design was conducted with 4 conditions and two words (trials) per condition was conducted. For details regarding the procedure and the conditions, please refer to experiment 2. Supplementary Table S2 shows the order of conditions for each subject. Note that, due to restrictions regarding the duration of the experiment in view of the COVID-19 pandemic, the think time was 45 s instead of 1 min.

### Scoring of the AUT

We used the fluency and flexibility sub-scores. The criteria to exclude incorrect responses was the same as experiment 1 and 2.

### Blink detection

The blink detection algorithm was similar to our previous studies (Brych & Händel, 2020; Brych et al., 2021; Murali & Händel, 2021) and same as in experiment 1.

### Results

#### Effect of movement and restriction on fluency

**Fig. 5:**
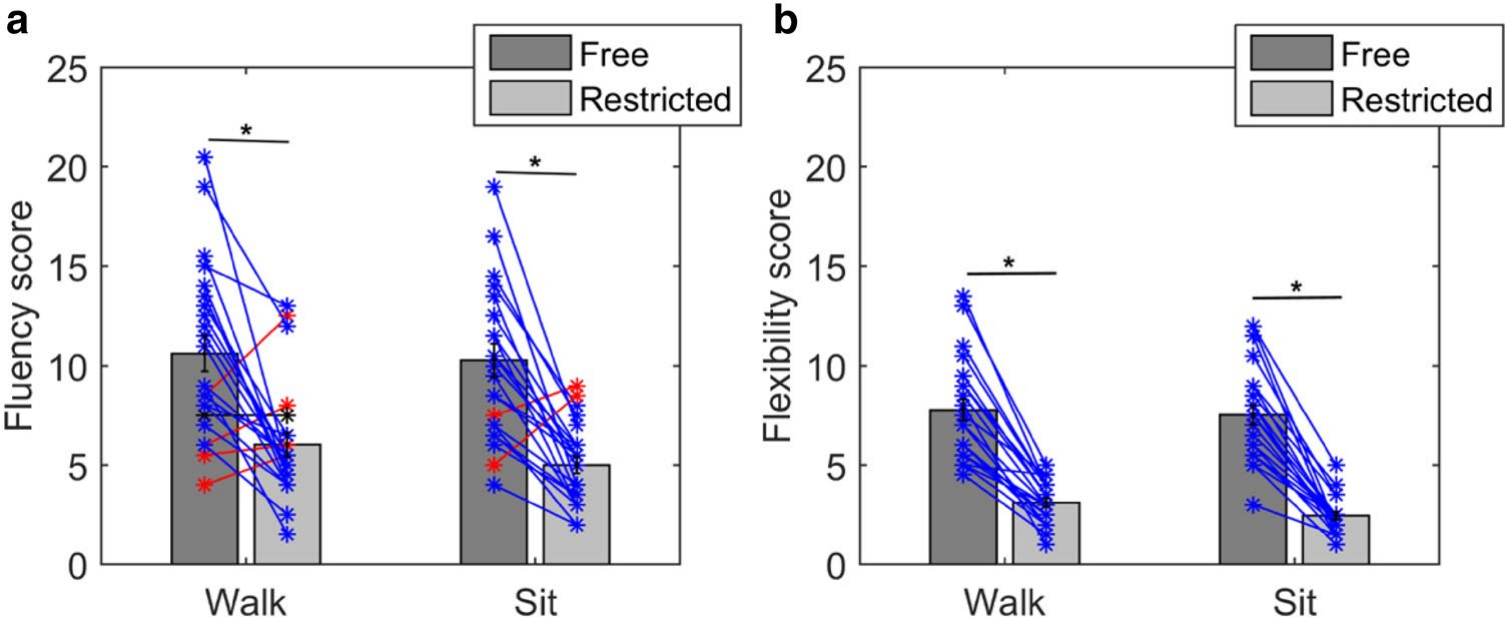
Mean fluency score (**a**) and flexibility score (**b**) during free and restricted walking and sitting in experiment 3 are shown. A two-factor ANOVA revealed a significant effect of restriction (*F*(1,91) = 39.9, *p* < 0.001), but no significant effect of movement (*F*(1,91) = 4.6, *p* = 0.0504). However, the *p* value for the factor movement was close to significance (0.0504). The asterisks represent data from individual subjects. The blue lines represent cases with higher scores during free movement, the red lines represent higher scores during restriction and black lines represent equal scores

The mean fluency score during free walking was 10.6 (SD = 4.2), during restricted walking was 6.02 (SD = 2.9), during free sitting was 10.3 (SD = 4.01) and during restricted sitting was 5 (SD = 2.03). The mean flexibility score during free walking was 7.8 (SD = 2.6), during restricted walking was 3.1 (SD = 1.04), during free sitting was 7.5 (SD = 2.4) and during restricted sitting was 2.4 (SD = 0.92). Figure 5 shows the mean scores in all conditions.

For fluency, a repeated-measures two-factor ANOVA was conducted between the factors Movement (Walk vs. Sit) and Restriction (free movement vs. restricted movement). The results showed a significant effect of restriction (*F*(1,91) = 39.9, *p* < 0.001), no significant effect of movement (*F*(1,91) = 4.6, *p* = 0.0504) and no significant interaction (*F*(1,91) = 2.6, *p* = 0.4). However, as one can see, the *p* value for the factor movement is close to significance (0.0504). A post-hoc *T* test for the factor Restriction (free movement vs. restricted movement) revealed that fluency scores were significantly higher during free movement *t*(45) = 7.8, *p* < 0.001).

For flexibility, a repeated-measures two-factor ANOVA was conducted between the factors Movement (Walk vs. Sit) and Restriction (free movement vs. restricted movement). The results showed a significant effect of restriction (*F*(1,91) = 170.2, *p* < 0.001), but not for movement (*F*(1,91) = 4.6, *p* = 0.07) and no significant interaction (*F*(1,91) = 0.8, *p* = 0.4). A post-hoc *T* test for the factor Restriction (free movement vs. restricted movement) revealed that flexibility scores were significantly higher during free movement (*t*(45) = 15.6, *p* < 0.001). In addition, supplementary Fig. S2 shows the mean fluency score over all subjects for each word. A within subject ANOVA revealed no significant effect of the words (*F*(7,179) = 0.7, *p* = 0.6).

#### Eye blinks related to different motor states and AUT scores

The mean think time blink rate during free walking was 15.4 (SD = 13.1) and during restricted walking was 16.8 (SD = 13.4). A *t* test showed no significant difference between the two conditions (*t*(21) = 1.6, *p* = 0.1). The mean time blink rate during free sitting was 12.1 (SD = 7.8) and during restricted sitting was 10.9 (SD = 10.6). A *t* test showed no significant difference between the two conditions (*t*(21) = 0.4, *p* = 0.6).

**Fig. 6:**
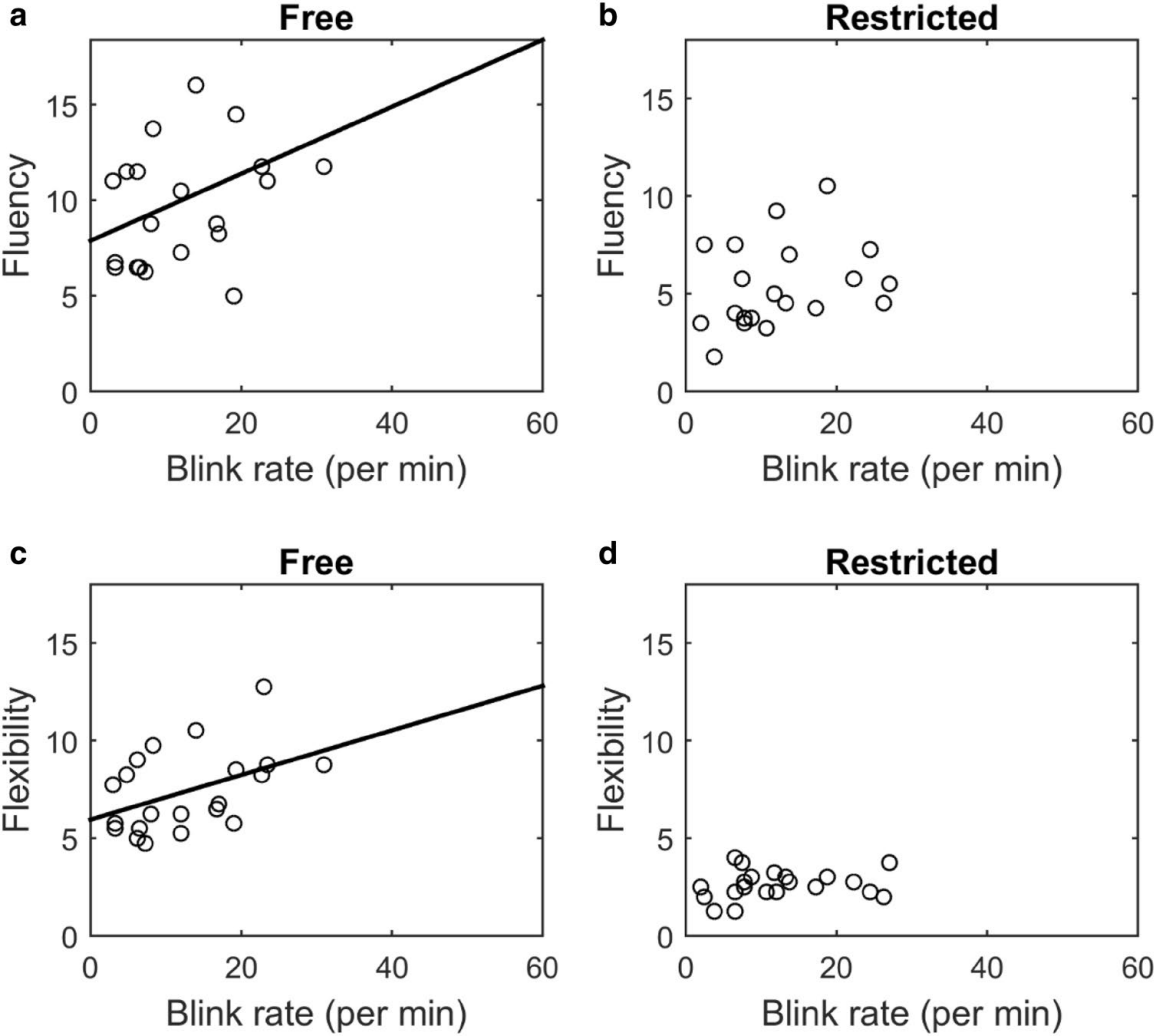
Mean fluency score (**a**, **b**) and flexibility scores (**c**, **d**) plotted against the blink rate for each subject during the free (**a**, **c**) and restricted (**b**, **d**) conditions are shown. Each circle represents data from a single subject. For fluency, a multiple regression model revealed a significant effect of blink rate (*F*(2,182) = 19.1, *p* < 0.001), no significant interaction between blink rate a restriction (*F*(2,182) = 3.8, *p* = 0.054) and no significant interaction between blink rate and movement (*F*(2,182) = 0.5, *p* = 0.4). The *p* value for the interaction between blink rate and restriction was close to significance (0.054). For flexibility, the model showed a significant effect of blink rate (*F*(1,182) = 18.4, *p* < 0.001), a significant interaction between blink rate a restriction (*F*(1,182) = 7.8, *p* = 0.01), but no significant interaction between blink rate and movement (*F*(1,182) = 0.3, *p* = 0.5)

To test if eye blink rates are linked to the scores, we conducted a multiple regression model with eye blink rate as a predictor and movement (walking or sitting) and restriction (free or restricted) as categorical independent variables. For fluency, the model revealed a significant effect of blink rate (*F*(2,182) = 19.1, *p* < 0.001), no significant interaction between blink rate and restriction (*F*(2,182) = 3.8, *p* = 0.054) and also no significant interaction between blink rate and movement (*F*(2,182) = 0.5, *p* = 0.4). The *p* value for the interaction between blink rate and restriction was close to significance (0.054). For flexibility, the model showed a significant effect of blink rate (*F*(1,182) = 18.4, *p* < 0.001), a significant interaction between blink rate and restriction (*F*(1,182) = 7.8, *p* = 0.01), but no significant interaction between blink rate and movement (*F*(1,182) = 0.3, *p* = 0.5). Figure 6 shows the mean scores plotted against blink rate, for each subject.

A post-Hoc regression analysis conducted on fluency vs. blink rate on the restricted and free conditions (taking the mean over the movement conditions) revealed no significance for free (*F*(1,20) = 3.5, r2 = 0.2, *p* = 0.07), or for the restricted (*F*(1,20) = 1.9, r2 = 0.1, *p* = 0.2) condition. A post-hoc regression test on flexibility revealed significance for the free (*F*(1,20) = 4.6, r2 = 0.2, *p* = 0.04) but not the restricted (*F*(1,20) = 0.7, r2 = 0.03, *p* = 0.4) condition.

Note that one outlier was excluded (due to blink rate > 3 standard deviations in the restricted sitting condition) in the above analysis (see Fig. 6).

In addition, to test for a possible within-subject effect of eye blinks on performance, we included a within subject analysis sorting trials as to low and high AUT scores for each subject and tested for a difference in blink rates. There was no indication of a relationship between blink rate and flexibility/fluency scores as can be seen in our Supplementary Material (see Fig. S3).

## Discussion

Our studies show that fluency and flexibility scores in a divergent thinking task are higher during unrestricted movement than during unrestricted movement. Although, previous work has already described a difference between free vs. restricted walking (Kuo & Yeh, 2016; Leung et al., 2012), we extend these findings by showing improved performance during free compared to restricted sitting, thereby identifying a movement state independent effect of restriction. We ascribe this effect to a broadening of the attentional focus, as will be discussed below. In addition, our data suggests that while the overall rate of spontaneous blinking can correlate with the performance in the AUT, i.e., subjects with a higher blink rate tend to score higher, a change in the blink rate within subjects does not go hand in hand with a modulation in performance. Accordingly, while we found that blink rates increase during other motor output, such as walking and speaking, this blink rate increase is not related to divergent thinking but likely depicts movement interaction as part of natural behaviour.

### The benefit of free movement during divergent thinking

The beneficial effect of free movement during divergent thinking seems surprising at first, since performance normally suffers during dual tasks. Activity involving gait or balance, indeed binds cognitive resources as they interfere with certain cognitive tasks (Al-Yahya et al., 2011; Patel et al., 2014). This contradiction was explained by the idea that, for certain tasks, such as the AUT, depleting cognitive resources might actually be beneficial by reducing top-down control mechanisms (Zhou et al., 2017). In fact, a study by Radel et al., (2015) showed that experimentally depleting cognitive resources through an additional task improved performance in the AUT. Both Radel et al., (2015) and Zhou et al., (2017) discuss the idea that a lack of top–down control leading to a decrease in attentional focus might benefit divergent thinking. This idea was applied to explain why participants performed better in a divergent thinking task while standing compared to while sitting or lying down (Zhou et al., 2017). Whereas in this case, it is easily conceivable that sitting or lying down required less cognitive resources, in our study, it is not obvious that following instructions during restriction would involve fewer cognitive resources than unrestricted movement. In fact, one might expect the opposite. Our finding that restricting and thereby controlling motor output has a negative effect on divergent thinking, therefore, rather supports an underlying mechanism of AUT improvement that is not based on the amount of cognitive or attentional resources but rather on the distribution of those resources. More specifically, we propose that the size of the attentional focus during free movement influences divergent thinking. Although, the influence of the size of attentional focus on creativity has been proposed before, as will be discussed in the next paragraph, the idea that the difference between free and restricted movement could stem from this attentional difference has not yet been put forward.

For quite some time, studies have shown that creative individuals tend to have a broader attentional focus than less creative individuals (Dykes & McGhie, 1976; Martindale, 1999; Mendelsohn & Griswold, 1964, 1966). More recent, studies have additionally revealed that manipulating attentional breadth can effect creative performance. For instance, Nijstad et al. (2010) conducted an experiment, wherein attentional breadth was manipulated via a Navon task (Navon, 1977). They found that attending to the larger letter during the task, improved performance in a subsequent AUT task. Similar results were also shown by Friedman et al. (2003), where participants completed a visual search task in a narrow or broad visual area. Specifically, the visual stimuli were present either in a narrow area around the fovea or at a comparably large area including the periphery. The concluded that after broadening the focus of visual attention, participants gave more original ideas in a subsequent AUT. According to the authors, the narrowing or broadening of perceptual (or visual) attention could facilitate the narrowing or broadening of conceptual attention. The benefit of a broad attentional focus on divergent thinking has been replicated (Memmert, 2007; Moraru et al., 2016) and the longitudinal study by Memmert (2007) could show that attention broadening training improved creative performance.

However, studies have not linked the positive effect of a broad attentional focus to the benefit of walking or free walking for divergent thinking. Here we propose that a main difference between restricted and unrestricted movement lies in the size of the focus of attention. During our restricted sitting condition, subjects had to fixate a small dot on the screen. This means the task directly implied a focus of attention on a very small area. With regard to free and restricted walking, a similar effect is likely, as subjects had to monitor the visual markers that defined their walking path. Indeed, walking itself seems to broaden the attentional focus as it has been shown that compared to standing still, walking leads to a stronger processing of peripheral visual input despite fixation (Cao & Händel, 2019). So even without explicit instruction, walking can broaden the attentional focus.

But how does a broad attentional focus facilitate divergent thinking? An explanation has been proposed by Nijastad in the dual pathway to creativity model (Nijstad et al., 2010). The dual pathway model proposes that two processes are involved in creative thinking, namely, cognitive flexibility and cognitive persistence. Flexibility involves switching between different ideas and concepts that are normally not associated with each other. On the other hand, persistence refers to the focused search for a specific solution. According to Nijstad et al. (2010) there could be a bias towards one or the other process, depending on the task. This idea has been extended to cognitive control by Hommel (2015) in the Metacontrol State Model (MSM). Both (Nijstad et al., 2010) and (Hommel, 2015) agree that a bias towards flexibility facilitates divergent thinking. Importantly, according to Nijstad et al. (2010), such a bias towards flexibility can be introduced by a broadening of attention.

To summarize, we propose that a broadening of the attentional focus during free compared to restricted movement biases the system towards flexibility and, therefore, improves divergent thinking. So rather than an activation of abstract concepts, such as voiced in the conceptual metaphor theory (Lakoff & Johnson, 1980) which was previously used to explain the positive effect of movement (Kuo & Yeh, 2016; Slepian & Ambady, 2012), we suggest that the attention based effect as previously described to influence divergent thinking can be extended to free movement.

### The relationship between eye blinks and divergent thinking

Can blink behaviour help us to assess a difference in attentional state during the various conditions of our creative thinking task? It has been shown that there is an active suppression of blinks during the processing of a visual stimulus (Bonneh et al., 2016; Murali & Händel, 2021; Wascher et al., 2015). This can be most prominently observed as blink rate reduction during the presentation of the sensory input and a subsequent blink rate increase (Brych & Händel, 2020; Oh et al., 2012; Siegle et al., 2008). The degree of blink rate modulation introduced by a stimulus is elevated if a perceptual task is included (Brych & Händel, 2020) and can further be suppressed by task difficulty (Oh et al., 2012). This could indicate that blinking is influenced by visual attentional load. When considering the difference between restricted and free conditions, subjects indeed needed to attend to the visual input that indicated the restriction (fixation point or marker for the walking path). However, the visual information was continuous, whereas the blink rate modulation is mostly observed around stimulus on- and offset. In addition, no task, such as a detection of temporal changes was required, which renders the suppression of blinks in order not to miss information unnecessary. It is, therefore, not surprising that we found no difference in blink rate between the restricted and free conditions (see Sect. 4.4.3). We conclude that the blink rate cannot shed further insights as to the attentional state during the two restriction conditions in our set up.

The comparably lower blink rate during sitting compared to walking (see Fig. 3) is likely based on movement interaction as part of natural behaviour. Eye blinks, like other movements, such as saccades and pupil size, are linked to other motor output, such as walking and speaking. For instance, blink rates have been reported to be higher during walking compared to standing (Cao & Händel, 2019), corresponding to the present results (see Fig. 3). Furthermore, we corroborated the finding of increased blink rates due to speaking (Brych et al., 2020; von Cramon & Schuri, 1980) which was clearly associated with the motor activity during speech production (Brych et al., 2020). Interestingly, we could show that this increase during speaking was specific to the sitting condition, while blink rates were similarly elevated during the walking condition for all three task phases (baseline, think time and response time). This suggests that blinking does not depict the quantitative motor output (as it is a non-cumulative effect) but rather marks a movement state. In other words, since walking and concurrent talking does not have a higher blink rate than walking or talking separately, the increase in eye blink rates goes hand in hand with a change in the state of movement, but more body parts moving would not lead to a stronger enhancement.

Overall, our data clearly shows that an increase in blinking, e.g., due to walking cannot be associated with an improvement in divergent thinking as restricted walking shows a clear blink rate increase but no behavioural improvement, while free sitting shows a low blink rate despite a behavioural improvement. In addition, within a movement condition, the blink rate does not indicate the ability for divergent thinking, as trials with high scores are not the ones with a high blink rate (see supplementary Fig. S3). This is true despite a quite sensitive within subject analysis.

However, we do find that subjects with a higher overall blink rate performed better in the AUT task during free movement. Previous studies have also shown such a between-subject correlation between eye blink rate and divergent thinking (Akbari Chermahini & Hommel, 2010; Ueda et al., 2016). While Akbari Chermahini and Hommel (2010) showed a quadratic relationship between baseline eye blink rates and AUT scores, Ueda et al. (2016) described a linear relationship when analysing eye blinks during the task. Baseline eye blink rates have been shown to reflect dopamine levels and, therefore, as Akbari Chermahini and Hommel (2010) put forth, benefit divergent thinking when they exhibit moderate rates. However, Ueda et al. (2016) explain the linear effect of task-related eye blinks through the activation of the default mode network. As mentioned in the introduction, the default mode network has been proposed to be involved during creativity (Beaty et al., 2014; Kühn et al., 2014). We can add, as the level of blink rate within a subject did not correlate with the scores (see Supplementary Fig. S3) a temporally fine-grained marker of dopamine is not likely. Our data further weakly indicates that blink rates mainly correlate during unrestricted behaviour. It is a possibility that only during unrestricted states the default mode network can exert its full influence. To our knowledge, studies have not tested the influence of restriction on the default mode network. However, as our study has a comparably low sample size compared to previous studies, future studies are required to test this specific effect.

## Summary

Our studies showed a beneficial effect of unrestricted (or free) movement on divergent thinking independent if restriction was minimized during walking or during sitting. We propose that this effect is due to a broadening of the field of attention when no restriction is placed. The subsequent benefit in divergent thinking might be introduced by a bias towards flexibility due to the broadened focus of attention (Nijstad et al., 2010). Our results further provide evidence that an externally guided increase in motor output, walking or blinking, will not lead to improved creativity, because if subjects are forced to move in a controlled fashion, they will not profit from it. These findings have several important implications. Given the current situation of the COVID-19 pandemic and the resulting increase in online teaching, it is important to understand its effects on learning and creativity. Since most online teaching involves fixating on a computer screen, the amount of free body movements, including head and eye movements, are greatly reduced compared to a normal classroom set up. Considering our findings, simple and effective strategies such as introducing periods of free movements in between sessions of online teaching, even during sitting, can improve the flow of ideas and aid in the learning process.

## Supplementary Information

Below is the link to the electronic supplementary material.

Supplementary file1 (DOCX 608 KB)

## References

[CR1] Akbari Chermahini S, Hommel B (2010). The (b) link between creativity and dopamine: Spontaneous eye blink rates predict and dissociate divergent and convergent thinking. Cognition.

[CR2] Al-Yahya E, Dawes H, Smith L, Dennis A, Howells K, Cockburn J (2011). Cognitive motor interference while walking: A systematic review and meta-analysis. Neuroscience & Biobehavioral Reviews.

[CR3] Beaty RE, Benedek M, Wilkins RW, Jauk E, Fink A, Silvia PJ, Hodges DA, Koschutnig K, Neubauer AC (2014). Creativity and the default network: A functional connectivity analysis of the creative brain at rest. Neuropsychologia.

[CR4] Blin O, Masson G, Azulay J, Fondarai J, Serratrice G (1990). Apomorphine-induced blinking and yawning in healthy volunteers. British Journal of Clinical Pharmacology.

[CR5] Bologna M, Fasano A, Modugno N, Fabbrini G, Berardelli A (2012). Effects of subthalamic nucleus deep brain stimulation and L-DOPA on blinking in Parkinson's disease. Experimental Neurology.

[CR6] Bonneh YS, Adini Y, Polat U (2016). Contrast sensitivity revealed by spontaneous eyeblinks: Evidence for a common mechanism of oculomotor inhibition. Journal of Vision.

[CR7] Brainard DH (1997). The psychophysics toolbox. Spatial Vision.

[CR8] Brych M, Händel B (2020). Disentangling top-down and bottom-up influences on blinks in the visual and auditory domain. International Journal of Psychophysiology.

[CR9] Brych M, Murali S, Händel B (2021). How the motor aspect of speaking influences the blink rate. PLoS One.

[CR10] Brych M, Murali S, Händel B (2021). The role of blinks, microsaccades and their retinal consequences in bistable motion perception. Frontiers in Psychology.

[CR11] Burr D (2005). Vision: In the blink of an eye. Current Biology.

[CR12] Cao L, Händel B (2019). Walking enhances peripheral visual processing in humans. PLoS Biology.

[CR13] Cao L, Chen X, Haendel BF (2020). Overground walking decreases alpha activity and entrains eye movements in humans. Frontiers in Human Neuroscience.

[CR14] Damiano C, Walther DB (2019). Distinct roles of eye movements during memory encoding and retrieval. Cognition.

[CR15] Dang LC, Donde A, Madison C, O'Neil JP, Jagust WJ (2012). Striatal dopamine influences the default mode network to affect shifting between object features. Journal of Cognitive Neuroscience.

[CR16] Dang, L. C., Samanez-Larkin, G. R., Castrellon, J. J., Perkins, S. F., Cowan, R. L., Newhouse, P. A., & Zald, D. H. (2017). Spontaneous eye blink rate (EBR) is uncorrelated with dopamine D2 receptor availability and unmodulated by dopamine agonism in healthy adults. *eneuro*, *4*(5). 10.1523/ENEURO.0211-17.2017 PMC5602106 28929131

[CR17] Doughty MJ (2001). Consideration of three types of spontaneous eyeblink activity in normal humans: During reading and video display terminal use, in primary gaze, and while in conversation. Optometry and Vision Science.

[CR18] Dykes M, McGhie A (1976). A comparative study of attentional strategies of schizophrenic and highly creative normal subjects. The British Journal of Psychiatry.

[CR20] Fogarty C, Stern JA (1989). Eye movements and blinks: Their relationship to higher cognitive processes. International Journal of Psychophysiology.

[CR21] Friedman RS, Fishbach A, Förster J, Werth L (2003). Attentional priming effects on creativity. Creativity Research Journal.

[CR22] Goldstein R, Bauer LO, Stern JA (1992). Effect of task difficulty and interstimulus interval on blink parameters. International Journal of Psychophysiology.

[CR23] Guilford, J. P. (1967). The nature of human intelligence.

[CR24] Hommel B (2015). Between persistence and flexibility: the Yin and Yang of action control. Advances in motivation science.

[CR25] Johansson R, Johansson M (2014). Look here, eye movements play a functional role in memory retrieval. Psychological Science.

[CR26] Johansson R, Holsanova J, Dewhurst R, Holmqvist K (2012). Eye movements during scene recollection have a functional role, but they are not reinstatements of those produced during encoding. Journal of Experimental Psychology: Human Perception and Performance.

[CR27] Kaminer J, Powers AS, Horn KG, Hui C, Evinger C (2011). Characterizing the spontaneous blink generator: An animal model. Journal of Neuroscience.

[CR28] Karson CN (1983). Spontaneous eye-blink rates and dopaminergic systems. Brain.

[CR29] Karson CN, Burns RS, LeWitt PA, Foster NL, Newman RP (1984). Blink rates and disorders of movement. Neurology.

[CR30] Kassner, M., Patera, W., & Bulling, A. (2014). Pupil: an open source platform for pervasive eye tracking and mobile gaze-based interaction. Proceedings of the 2014 ACM international joint conference on pervasive and ubiquitous computing: Adjunct publication.

[CR31] Kleiner, M., Brainard, D., & Pelli, D. (2007). What's new in Psychtoolbox-3?

[CR32] Kühn S, Ritter SM, Müller BC, Van Baaren RB, Brass M, Dijksterhuis A (2014). The importance of the default mode network in creativity—a structural MRI study. The Journal of Creative Behavior.

[CR33] Kulisevsky J, Pagonabarraga J, Martinez-Corral M (2009). Changes in artistic style and behaviour in Parkinson’s disease: Dopamine and creativity. Journal of Neurology.

[CR34] Kuo C-Y, Yeh Y-Y (2016). Sensorimotor-conceptual integration in free walking enhances divergent thinking for young and older adults. Frontiers in Psychology.

[CR35] Lakoff, G., & Johnson, M. (1980). Metaphor we live by. *Chicago/London*.

[CR36] Lenoble Q, Janssen SM, El Haj M (2019). Don’t stare, unless you don’t want to remember: Maintaining fixation compromises autobiographical memory retrieval. Memory.

[CR37] Leung AK-Y, Kim S, Polman E, Ong LS, Qiu L, Goncalo JA, Sanchez-Burks J (2012). Embodied metaphors and creative “acts”. Psychological Science.

[CR38] Madore KP, Addis DR, Schacter DL (2015). Creativity and memory: Effects of an episodic-specificity induction on divergent thinking. Psychological Science.

[CR39] Martindale, C. (1999). Biological bases of creativity.

[CR40] Mednick S (1962). The associative basis of the creative process. Psychological Review.

[CR41] Memmert D (2007). Can creativity be improved by an attention-broadening training program? An exploratory study focusing on team sports. Creativity Research Journal.

[CR42] Mendelsohn GA, Griswold BB (1964). Differential use of incidental stimuli in problem solving as a function of creativity. The Journal of Abnormal and Social Psychology.

[CR43] Mendelsohn GA, Griswold BB (1966). Assessed creative potential, vocabulary level, and sex as predictors of the use of incidental cues in verbal problem solving. Journal of Personality and Social Psychology.

[CR44] Moraru A, Memmert D, van der Kamp J (2016). Motor creativity: The roles of attention breadth and working memory in a divergent doing task. Journal of Cognitive Psychology.

[CR45] Murali S, Händel B (2021). The latency of spontaneous eye blinks marks relevant visual and auditory information processing. Journal of Vision.

[CR46] Nagano-Saito A, Liu J, Doyon J, Dagher A (2009). Dopamine modulates default mode network deactivation in elderly individuals during the Tower of London task. Neuroscience Letters.

[CR47] Nakano T, Kato M, Morito Y, Itoi S, Kitazawa S (2013). Blink-related momentary activation of the default mode network while viewing videos. Proceedings of the National Academy of Sciences.

[CR48] Navon D (1977). Forest before trees: The precedence of global features in visual perception. Cognitive Psychology.

[CR49] Nijstad B, De Dreu C, Rietzschel E, Baas M (2010). The dual pathway to creativity model: Creative ideation as a function of flexibility and persistence. European Review of Social Psychology.

[CR50] Oh J, Jeong S-Y, Jeong J (2012). The timing and temporal patterns of eye blinking are dynamically modulated by attention. Human Movement Science.

[CR51] Oppezzo M, Schwartz DL (2014). Give your ideas some legs: The positive effect of walking on creative thinking. Journal of Experimental Psychology: Learning, Memory, and Cognition.

[CR52] Ottemiller DD, Elliott CS, Giovannetti T (2014). Creativity, overinclusion, and everyday tasks. Creativity Research Journal.

[CR53] Patel P, Lamar M, Bhatt T (2014). Effect of type of cognitive task and walking speed on cognitive-motor interference during dual-task walking. Neuroscience.

[CR54] Pelli DG (1997). The VideoToolbox software for visual psychophysics: Transforming numbers into movies. Spatial Vision.

[CR55] Radel R, Davranche K, Fournier M, Dietrich A (2015). The role of (dis) inhibition in creativity: Decreased inhibition improves idea generation. Cognition.

[CR56] Runco MA, Acar S (2012). Divergent thinking as an indicator of creative potential. Creativity Research Journal.

[CR57] Salvi C, Bowden EM (2016). Looking for creativity: Where do we look when we look for new ideas?. Frontiers in Psychology.

[CR59] Sescousse G, Ligneul R, van Holst RJ, Janssen LK, de Boer F, Janssen M, Berry AS, Jagust WJ, Cools R (2018). Spontaneous eye blink rate and dopamine synthesis capacity: Preliminary evidence for an absence of positive correlation. European Journal of Neuroscience.

[CR60] Siegle GJ, Ichikawa N, Steinhauer S (2008). Blink before and after you think: Blinks occur prior to and following cognitive load indexed by pupillary responses. Psychophysiology.

[CR61] Slepian ML, Ambady N (2012). Fluid movement and creativity. Journal of Experimental Psychology: General.

[CR62] Slepian ML, Ambady N (2014). Simulating sensorimotor metaphors: Novel metaphors influence sensory judgments. Cognition.

[CR64] Strakowski SM, Sax KW (1998). Progressive behavioral response to repeated d-amphetamine challenge: Further evidence for sensitization in humans. Biological Psychiatry.

[CR65] Strakowski SM, Sax KW, Setters MJ, Keck PE (1996). Enhanced response to repeated d-amphetamine challenge: Evidence for behavioral sensitization in humans. Biological Psychiatry.

[CR66] Taylor J, Elsworth J, Lawrence M, Sladek J, Roth R, Redmond D (1999). Spontaneous blink rates correlate with dopamine levels in the caudate nucleus of MPTP-treated monkeys. Experimental Neurology.

[CR67] Ueda Y, Tominaga A, Kajimura S, Nomura M (2016). Spontaneous eye blinks during creative task correlate with divergent processing. Psychological Research Psychologische Forschung.

[CR68] von Cramon D, Schuri U (1980). Blink frequency and speech motor activity. Neuropsychologia.

[CR69] Wascher E, Heppner H, Möckel T, Kobald SO, Getzmann S (2015). Eye-blinks in choice response tasks uncover hidden aspects of information processing. EXCLI Journal.

[CR70] Zabelina DL, Colzato L, Beeman M, Hommel B (2016). Dopamine and the creative mind: Individual differences in creativity are predicted by interactions between dopamine genes DAT and COMT. PLoS ONE.

[CR71] Zhang W, Sjoerds Z, Hommel B (2020). Metacontrol of human creativity: The neurocognitive mechanisms of convergent and divergent thinking. NeuroImage.

[CR72] Zhou Y, Zhang Y, Hommel B, Zhang H (2017). The impact of bodily states on divergent thinking: Evidence for a control-depletion account. Frontiers in Psychology.

