# Supplementary material for: Motor restrictions impair divergent thinking during walking and during sitting
Source: Psychol Res. 2022 Jan 8;86(7):2144–57. doi: 10.1007/s00426-021-01636-w (PMC8742166; doi:10.1007/s00426-021-01636-w)
Supplement: Supplementary file 1 — Supplementary file1 (DOCX 608 KB) [file 426_2021_1636_MOESM1_ESM.docx]

**Motor restrictions impair divergent thinking during walking and during sitting**

Supriya Murali^1*^, Barbara Händel^1^

^1^ Institute of Psychology III, University of Würzburg

*** Corresponding author:**Supriya Murali
Email: [supriya.murali@uni-wuerzburg.de](mailto:supriya.murali@uni-wuerzburg.de)

**Supplementary Material**

**
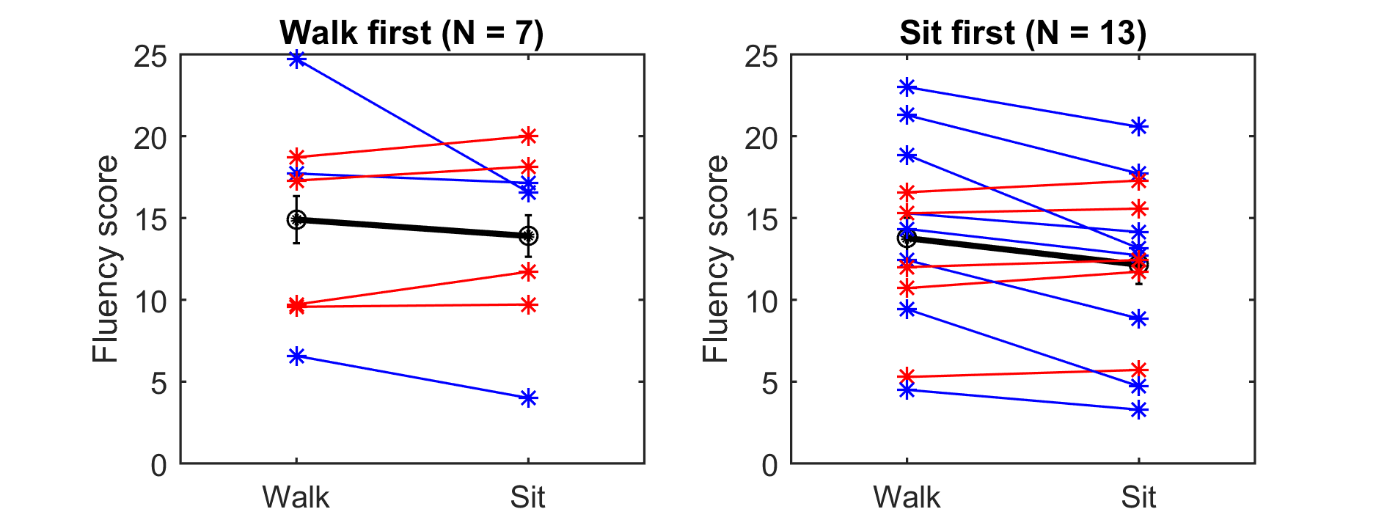
**

**Figure S1** Shows the mean fluency scores for walking and sitting dependent on the order of the conditions in experiment 1; difference between the walk and sit condition for those who did the walk condition first (left) and those who did the sit condition first (right). The asterisks represent data from each subject. The blue lines represent cases where the fluency score is higher for the walking condition and the red lines represent cases wherein it is higher for the sitting condition. A t-test showed significance only for those who sat first (t (12) = 2.6, p = .02) and no significance for those who walked first (t (6) = 0.7, p = .5).

**
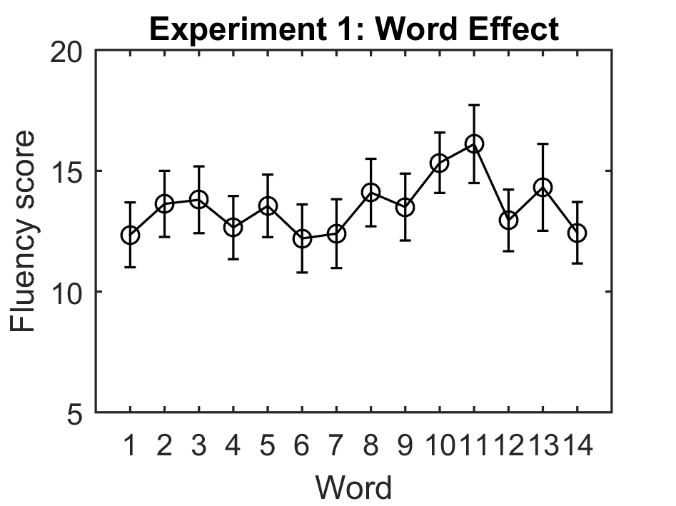

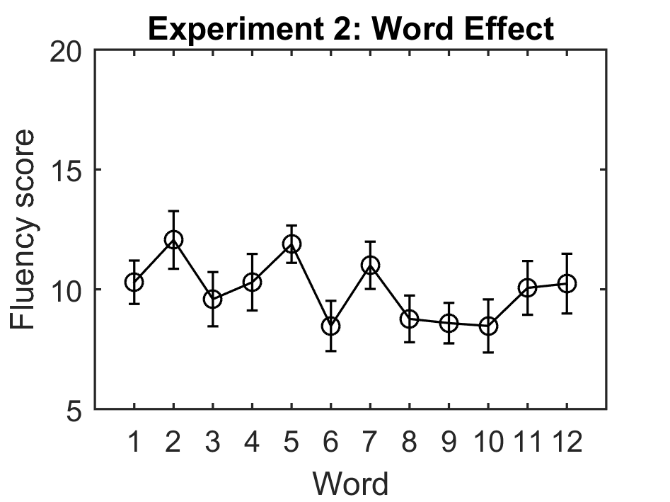
**

**
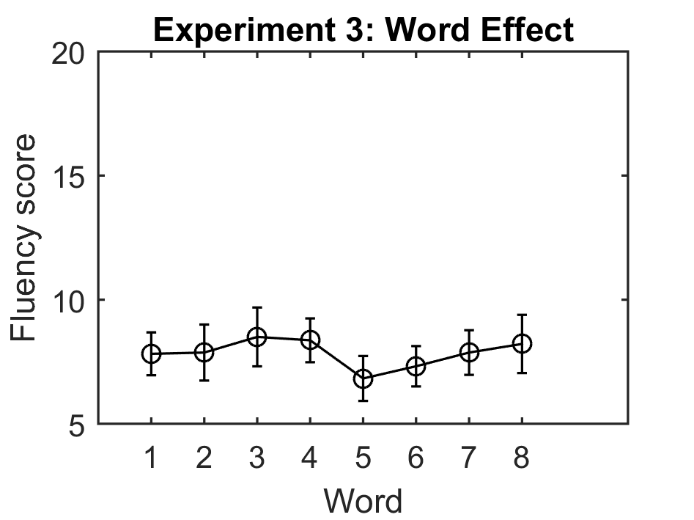
**

**Figure S2** shows the mean fluency score over all subjects for each of the 14 words in experiment 1 and 12 words in experiment 2. An ANOVA revealed no effect of the words in experiment 1 (F (13,269) = 0.96, p = .5), experiment 2 (F (11,203) = 0.3, p = .9) and experiment 3 (F (7,179) = 0.7, p = .6)

**Table S1**

***Shows the order of the conditions for each subject of experiment 2.***


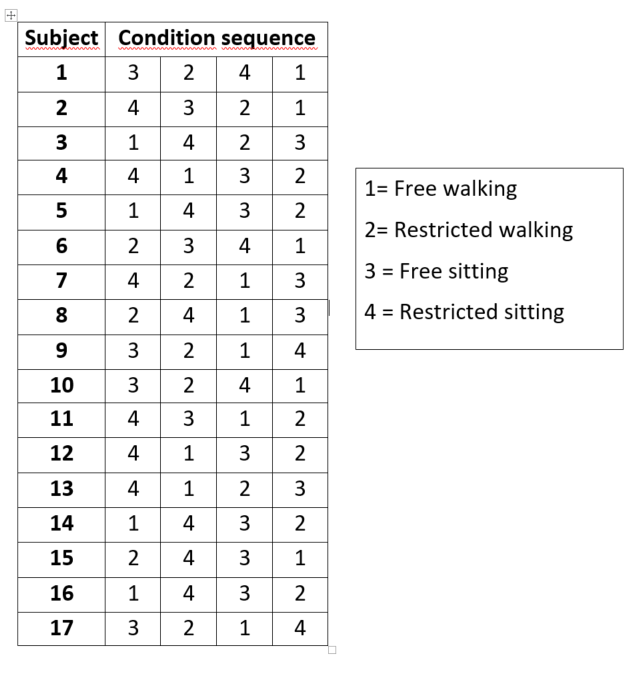


**Table S2**

***Shows the order of the conditions for each subject of experiment 3.***

***
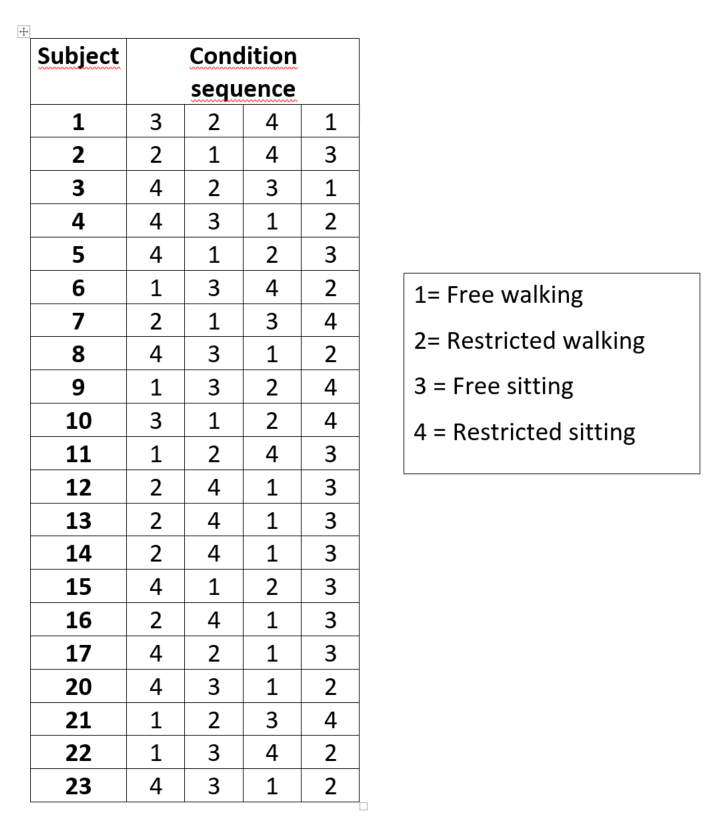
***

To exclude any within subject influence, we took the difference between the blink rates of the trials with lowest and highest fluency for each subject in experiment 1 and 3 and conducted a one-sample T-test against 0 (see Figure 15). The T-test found no significant difference in walking (t(40) = .6, p = .5) or for sitting (t(40) = 1.5, p = .1). The same was done using the flexibility scores which also showed no significant difference during walking (t(40) = 1.5, p = .1) or during sitting (t(40) = 1.1, p = .2).


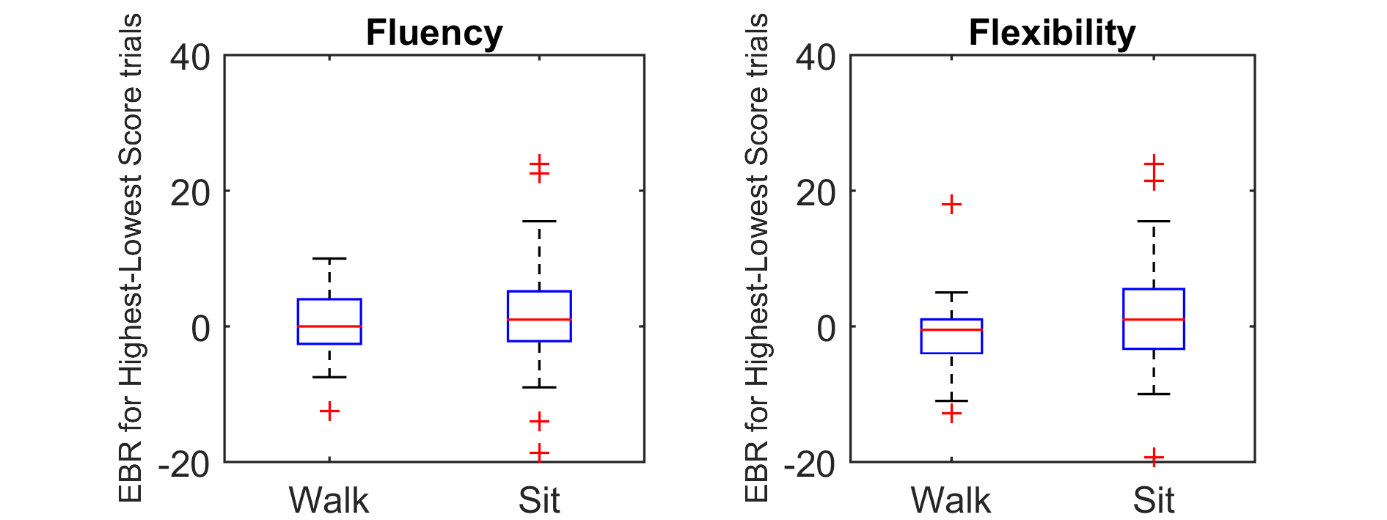


**Figure S3.** shows the difference in eye blink rate of the highest and lowest fluency (left) and flexibility (right) scores during walking and during sitting. The T-test found no significant difference from zero during walking (t(40) = .6, p = .5) or for sitting (t(40) = 1.5, p = .1) when taking fluency scores. Similarly, no significant difference was found during walking (t(40) = 1.5, p = .1) or during sitting (t(40) = 1.1, p = .2) when taking flexibility scores.
